# Application of thermo-cylindrical type focused-ultrasound as novel milk pasteurization: microbial inactivation, immunoglobulin G retention, and physicochemical characteristics

**DOI:** 10.1016/j.ultsonch.2025.107615

**Published:** 2025-10-23

**Authors:** Prabhathma Yasasvi Rathnayake, Soyeong Kim, Rina Yu, Chemin Nam, Sin-Young Park, Seonae Hwangbo, Hae In Yong

**Affiliations:** a Department of Animal Science and Biotechnology, Chungnam National University, Daejeon 34134, Republic of Korea; b R&D Center, FUST Lab Co., Ltd., Daejeon 34015, Republic of Korea; c Department of Animal Resources Science, Kongju National University, Chungnam 32439, Republic of Korea

**Keywords:** Focused-ultrasound, LTLT, Milk pasteurisation, Milk safety, Milk quality

## Abstract

This study aimed to investigate the efficacy of focused ultrasound as a novel milk pasteurisation technology to replace conventional low-temperature long-time (LTLT) method. The microbial inactivation, immunoglobulin G (IgG) retention, and physicochemical properties of milk treated with focused ultrasound were evaluated. Thermo-cylindrical type (55 °C) focused ultrasound was used for all experiments. Milk was subjected to focused ultrasound at 30 W and 100 W for 0, 10, 20, and 30 min to investigate the antimicrobial effects of this method. The total aerobic bacteria, coliforms, *Escherichia coli* O157:H7, and *Listeria monocytogenes* counts decreased (p < 0.05) with increasing focused-ultrasound power and treatment time. Thus, focused ultrasound treatments were applied at 30 W and 100 W for 30 min in subsequent milk quality evaluation and compared with LTLT-treated milk. The IgG retention value of milk treated with focused ultrasound at 30 W and 100 W was higher than that of LTLT-treated milk (p < 0.05). The alkaline phosphatase activity, pH, and whiteness index of milk treated with focused ultrasound at 100 W did not differ from LTLT-treated milk (p > 0.05). Moreover, milk treated with focused ultrasound at 100 W exhibited the smallest particle size and fat globules (p < 0.05). In conclusion, focused-ultrasound treatment at 55 °C and 100 W for 30 min improves milk microbial safety. Additionally, it preserves IgG activity and improves milk quality and stability by reducing fat globule size. Thus, focused ultrasound has the potential to become a novel milk pasteurisation technology for excellent antimicrobial effects and milk quality.

## Graphical abstract

**Figure:**
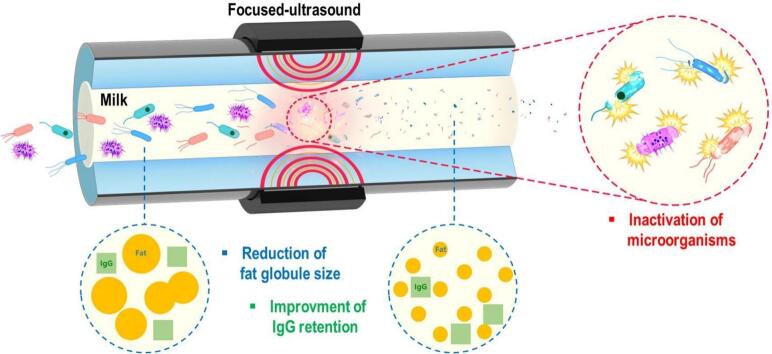

## 1 Introduction

Milk is considered a complete food because of its rich and balanced composition of macronutrients (fats, proteins, and carbohydrates), essential vitamins (vitamins A, D, and B_12_), and minerals (calcium, potassium, and phosphorus) [1]. In addition, milk contains several bioactive components that contribute to immune modulation and support intestinal health, including immunoglobulins, lactoferrin, lactadherin, and oligosaccharides. Thus, milk is an important dietary component for individuals across all age groups [2].

Despite its benefits, global milk consumption per capita is declining. Global milk consumption per person declined by 0.2 % in 2022, indicating a notable downward trend in milk consumption worldwide [3]. Thus, the dairy industry needs to develop new products and marketing strategies to boost consumer appeal and reverse the downward trend in milk consumption. According to worldwide consumer dietary patterns, health and wellness considerations have become a major concern for consumers when making food choices in recent years, and this trend is expected to increase by 10 % by 2030 [4]. Therefore, one approach for enhancing the dairy industry is to produce healthier milk by preserving its nutrient content and health components, such as bioactive compounds (enzymes, vitamins, and immunoglobulins that provide health benefits), along with fulfilling food safety standards for microbial inactivation in milk [5].

In the current dairy industry, the low-temperature long-time pasteurisation method (LTLT; 63 °C, 30 min) effectively inactivates microorganisms and extends the shelf life of milk at its lowest temperature [6]. Despite its effectiveness in microbial inactivation, this method has several disadvantages because of its temperature, including protein denaturation, degradation of heat-sensitive vitamins, and the development of off-flavours [6,7]. Additionally, LTLT causes the denaturation of immunoglobulin G (IgG) in cow milk [8]. Therefore, a lower-temperature pasteurisation technology (compared to LTLT) is required for better preservation of the heat-labile nutrients and bioactive compounds in milk while still achieving microbial inactivation.

One promising alternative is ultrasound pasteurisation technology, which has demonstrated the potential for effective microbial inactivation without undesirable milk quality changes [7]. Furthermore, ultrasound with mild heat can effectively enhance microbial inactivation while minimising the thermal degradation of milk components [7,9]. Ultrasound-induced acoustic cavitation inactivates microbes in milk via physical and chemical mechanisms. Acoustic waves generate microbubbles that collapse violently during compression-rarefaction cycles, consequently producing localised shock waves, high temperatures, and pressures that disrupt microbial cells [9]. This collapse also creates shear forces, microjets, and turbulence, thereby physically damaging cell membranes and intracellular structures. Chemically, cavitation homolyses water vapour into reactive oxygen species (•OH and H_2_O_2_), resulting in oxidative stress [10]. Together, these effects induce membrane rupture, leakage of cellular components, and degradation of DNA and proteins, leading to microbial inactivation.

Ultrasound technology generally utilises bath-type or horn-type sonication systems. However, these systems have several limitations, including non-uniform energy distribution, low energy efficiency, and non-adjustable frequency and power [11,12]. To address these drawbacks, the focused-ultrasound system has emerged as an advanced alternative. In particular, cylindrical focused ultrasound systems are employed to emit ultrasound energy uniformly in all 360° directions, consequently ensuring a homogeneous energy distribution across the sample. Additionally, the design allows for the concentration and amplification of energy at the centre of the system, thereby enhancing the treatment efficiency. Moreover, the incorporation of a circulating cooling water system enables precise temperature control (high or low temperatures); this facilitates continuous operation and improves the dispersion performance [11,12]. However, to date, no studies have investigated the combined application of cylindrical-focused ultrasonic systems and mild heat for microbial inactivation in milk.

Therefore, in the present study, we aimed to determine the optimal power and time conditions for milk pasteurisation using a thermo-cylindrical focused-ultrasound system at 55 °C. The effect of focused ultrasound on the inactivation of pathogenic and hygienic indicator microorganisms was also evaluated. Furthermore, IgG retention and physicochemical properties of milk were investigated using a focused ultrasound system and compared with those of conventional LTLT pasteurisation.

## 2 Materials and methods

### 2.1 Milk sample collection and handling

Raw cow milk was obtained from a local farm (Seonchang farm, Chungnam, Republic of Korea) to analyse hygienic indicator microorganisms (total aerobic bacteria and coliforms) and physicochemical properties. The raw milk was collected in sterile, insulated containers and transported under refrigerated conditions (4 ± 2 °C). Upon arrival at the laboratory, they were stored at 4 °C until further analysis.

Commercial sterilised milk (processed at 135 °C for 3 s) was purchased from a local retailer to evaluate the inactivation of pathogens (*Escherichia coli* O157:H7 and *Listeria monocytogenes)*. The use of sterilised milk, which is free from native microorganisms, allows for the accurate evaluation of focused ultrasound efficacy against inoculated pathogens. The sterilised milk was stored at 4 °C before the inoculation test.

### 2.2 Application of focused ultrasound and heat treatments

A thermo-cylindrical focused ultrasound system (DEBREX; FUST Lab Co., Ltd., Daejeon, Republic of Korea) equipped with a single-lead zirconate titanate transducer was used to deliver uniform, high-energy sonication. The power output of the device was set to either 30 or 100 W for separate experimental runs. Milk was introduced into the focused ultrasound device at a constant flow rate of 20 mL/min and sonicated. The treatment times were 10, 20, and 30 min for the microbial analysis and 30 min for the physicochemical analysis under each power setting (30 and 100 W). The system has a cylindrical double-jacket structure, in which the milk flows through the central channel while water circulates through the outer jacket. During treatment, the temperature of the milk was continuously monitored and maintained at 55 ± 2 °C using a circulating water bath. Following sonication, the milk samples were immediately refrigerated at 4 °C to rapidly cool.

Because the focused ultrasound treatment was conducted at 55 °C, a heat control (55 °C) was also used as treatment to distinguish thermal effects in the microbial inactivation and physicochemical changes of milk. The heat control sample was subjected to a focused ultrasound system, and it was treated at 55 °C for 10, 20, and 30 min without ultrasound application.

LTLT treatment (63 °C, 30 min) was used as a positive control in the present study. For LTLT, the milk was heated in a water bath (HB-205SW; Hanbeak Scientific Technology, Gyeonggi-do, Republic of Korea) at 63 °C for 30 min [13]. As LTLT is a conventional milk pasteurisation technology, it was used only for physicochemical analyses and not for microbiological evaluations.

#### 2.2.1 Calculation of delivered sonic energy (DSE)

Ultrasonication dosage was quantified as delivered sonic energy (DSE, J/mL), which represents the actual ultrasonic energy delivered to the sample. The DSE was calculated according to the calorimetric method as follows:

(1) $$P=\left(\frac{\mathrm{dT}}{\mathrm{dt}}\right)\times M\times C_{p}$$

(2) $$\mathrm{DSE}=\frac{P\times t}{V}$$

where *P* is the delivered ultrasonic power (J/s), *dT/dt* is the initial temperature-rise slope (K/s), *M* is the sample mass (g), *C_p_* is the specific heat capacity of milk (approximated as 3.9 Jg^-1^K^−1^), *t* is the treatment time (s), and *V* is the sample volume (mL) [14]. The sample temperature was maintained using a circulating water bath. Therefore, a continuous dT/dt could not be obtained, and the delivered power was estimated from the initial 20 s temperature-rise slope (short adiabatic calorimetric run), during which heat loss can be considered negligible. The operating frequency of the focused ultrasound system was approximately 380 kHz for both 30 W and 100 W treatments. Based on a treatment time of 30 min with a 50 mL milk sample, the calculated DSE values were approximately 1,550 J/mL at 30 W and 2,920 J/mL at 100 W.

### 2.3 Evaluation of microbial inactivation in milk

#### 2.3.1 Pathogen inoculation procedure

*E. coli* O157:H7 (NCCP 17169) was cultured in tryptic soy broth (TSB; MB Cell, Seoul, Republic of Korea), whereas *L. monocytogenes* (ATCC 19111) was cultured in TSB supplemented with 0.6 % yeast extract (Thermo Fisher Scientific, Waltham, MA, USA). Cultures were incubated at 37 °C for 48 h. Following incubation, the cultured bacteria were transferred into 50-mL centrifuge tubes and centrifuged at 4000 rpm for 20 min at 4 °C using a refrigerated centrifuge (1580R; Labogene, Gyrozen Co., Ltd., Gimpo, Republic of Korea). The resulting pellets were resuspended in sterile saline (0.85 %) to obtain a final suspension concentration of approximately 10^6^-10^7^ CFU/mL of suspension. Subsequently, 500 µL of each bacterial suspension (*E. coli* O157:H7 and *L. monocytogenes*) was inoculated into 50 mL of sterilised commercial milk. The inoculated milk samples were then treated with focused ultrasound and heat control.

#### 2.3.2 Microbial analysis

Following focused ultrasound treatments, milk samples were serially diluted in a sterile 0.85 % saline solution. Subsequently, 100  µL of each dilution was spread into agar media, depending on the target microorganism. *E. coli* O157:H7, *L. monocytogenes*, total aerobic bacteria, and coliforms were respectively spread on tryptic soy agar (TSA; MB Cell, Seoul, Republic of Korea), TSA supplemented with 0.6 % yeast extract (Thermo Fisher Scientific, Waltham, MA, USA), plate count agar (PCA; MB Cell, Seoul, Republic of Korea), and eosin methylene blue agar (EMB; MB Cell, Seoul, Republic of Korea). All plates were incubated at 37 °C for 24 h. After incubation, the colonies were counted and expressed as log colony-forming units per millilitre (Log CFU/mL).

### 2.4 Evaluation of physicochemical properties

#### 2.4.1 Alkaline phosphatase (ALP) activity

The activity of ALP was measured as described by Shamsi et al. [15], with slight modifications. An ALP yellow liquid substrate system (P7998; Sigma-Aldrich, St Louis, MO, USA) containing p-nitrophenyl phosphate was used. An ALP assay buffer solution was prepared by dissolving 1.5 g/L NaHCO_3_ and 3.5 g/L anhydrous Na_2_CO_3_ in distilled water. The pH of the buffer was adjusted to 10.2 using 1 mol/L NaOH. For sample preparation, milk samples were diluted 1:50 (v/v) in buffer solution. Subsequently, 500 μL of the ALP liquid substrate system was added to 1 mL microcentrifuge tubes and incubated at 37 °C for 5 min in a water bath (HB-205SW; Hanbeak Scientific Technology, Gyeonggi-do, Republic of Korea). Then, 100 μL of each diluted milk sample was added to the respective microcentrifuge tubes containing the substrate, and the tubes were vortexed briefly to mix. The microcentrifuge tubes were incubated at 37 °C for 5 min and transferred to an ice bath for 5 min to stop the enzymatic reaction. The contents of each tube were then pipetted into a 96-well microtest plate for absorbance measurements at 410 nm using a multimode microplate reader (Varioskan Lux; Thermo Fisher Scientific, MA, USA) within 2–3 min. The absorbance values were used to calculate the concentration of p-nitrophenol (p-NP) in each milk sample based on a standard curve constructed using p-NP solutions at concentrations of 0, 5, 10, 15, 20, and 25 µg/mL in Na_2_CO_3_ buffer. One unit of ALP activity was defined as the release of 1 µmol/L of p-NP per minute at pH 10.2 and 37 °C.

#### 2.4.2 IgG retention

The concentration of IgG in milk samples was quantified using a commercially available enzyme-linked immunosorbent assay kit (Bethyl Laboratories Inc., Fortis Life Sciences, Boston, MA, USA) following the manufacturer's protocol. Briefly, milk samples were diluted to a ratio of 1:50,000 (v/v) using a dilution buffer. Subsequently, 100 µL of the diluted sample was added to designated wells of a 96-well microplate, which was then covered and incubated at room temperature (20–25 °C) for 1 h. After incubation, the wells were washed four times with washing buffer. Then, 100 µL of the anti-IgG detection antibody was added to each well and incubated at room temperature for another hour, followed by a second washing step. Next, 100 µL of horseradish peroxidase solution was added to each well and incubated for 1 h at room temperature, followed by another wash cycle. Subsequently, 100 µL of TMB (3,3′,5,5′-tetramethylbenzidine) substrate solution was added to each well, and the plate was incubated in the dark at room temperature for 30 min. The enzymatic reaction was terminated by adding 100 µL of stop solution to each well. The absorbance was measured at 450 nm using a multimode microplate reader (Varioskan Lux; Thermo Fisher Scientific, MA, USA). The IgG concentrations in the milk samples were determined based on a standard curve generated using known IgG standards in accordance with the manufacturer’s instructions. Subsequently, the percentage of IgG retained in the milk samples after treatment was calculated based on the IgG concentration in raw milk.

#### 2.4.3 Particle size and particle size distribution

The particle size and particle size distribution of the milk samples were measured using a particle analyser (Litesizer 500; Anton Paar GmbH, Graz, Austria). Milk samples were diluted in ultrapure water at a ratio of 1:200 (v/v). Then, a 1.2-mL aliquot of the diluted milk was transferred to a cuvette, and particle size values were recorded according to the manufacturer's standard operating procedures. Particle size was expressed as the D_50_ value (median particle size), representing the central tendency of particle size distribution [16], and the D_32_ value (Sauter mean diameter), which reflects the surface-area-weighted mean diameter of droplets [17]. The D_32_ value was calculated using Equation 3.

(3) $$D_{32}=\frac{\sum n_{i}d_{i}^{3}}{\sum n_{i}d_{i}^{2}}$$

#### 2.4.4 Zeta potential

The zeta potential of the milk samples was measured using a particle analyser (Litesizer 500; Anton Paar GmbH, Graz, Austria) based on electrophoretic light scattering. Initially, milk samples were diluted with ultrapure water at a ratio of 1:20 (v/v). Then, a 5 mL aliquot of the diluted milk was transferred into a cuvette, and zeta potential values were recorded as described by Scudino et al. [7].

#### 2.4.5 Turbidity

The turbidity of the milk samples was assessed based on the absorbance values as described by Shanmugam et al. [18], with slight modifications. Milk samples were initially diluted with distilled water at a ratio of 1:50 (v/v) to ensure optimal spectrophotometric analysis, and absorbance was measured at 860 nm using a UV–VIS spectrophotometer (Ubi 490; MicroDigital Co., Ltd., Seoul, Republic of Korea).

#### 2.4.6 Microstructure of the fat globules

The microstructure of milk fat globules was analysed using fluorescence and optical microscopy as described by Argov-Argaman et al. [19] with slight modifications. Milk samples were diluted with distilled water at a ratio of 1:50 (v/v), and 100 µL of the diluted sample was stained with Nile red (Sigma-Aldrich, MO, USA) in ethanol (42 µg/mL). The stained samples were then incubated at room temperature for 2 h before imaging. Microscopic analyses were performed using an Olympus BX-53 microscope (Olympus Optical Co., Ltd., Tokyo, Japan) equipped with a fluorescent filter. The acquired images were processed and analysed using image analysis software (Cell*; Soft Imaging System, Münster, Germany).

#### 2.4.7 Colour values

The colour properties of raw and focused ultrasound-treated milk samples were evaluated using a colorimeter (CM-5; Konica Minolta, Osaka, Japan) based on the International Commission on Illumination (CIE) parameters: L* (+ lightness, − darkness), a* (+ redness, − greenness), and b* (+ yellowness, − blueness). The colorimeter was calibrated using a standard black plate and distilled water before the analysis. For each measurement, 2 mL of the milk sample was used to determine the colour attributes. The total colour difference (ΔE) and whiteness index were then calculated according to Equations 4 and 5, respectively, as described by Milovanovic et al. [20].

(4) $${\Delta E}^{}=\sqrt{{\left(\Delta L^{*}\right)}^{2}+{\left(\Delta a^{*}\right)}^{2}+{\left(\Delta b^{*}\right)}^{2}}$$

(5) $$\mathrm{Whitnessindex}=100-\sqrt{{{(100-L}^{*})}^{2}+{{(a}^{*})}^{2}+{{(b}^{*})}^{2}}$$

#### 2.4.8 pH

The pH of milk samples was measured at room temperature using a calibrated pH meter (PM-3; CAS Co., Ltd., Seoul, Republic of Korea). Milk samples were briefly vortexed before measurement, and the pH meter probe was calibrated with standard buffer solutions of pH 7.0, 4.01, and 10.0 before use to collect accurate readings.

#### 2.4.9 Thiobarbituric acid reactive substances (TBARS)

Lipid peroxidation in milk samples was assessed by measuring the malondialdehyde (MDA) concentration using the TBARS assay. The analysis followed the spectrophotometric method described by Ohkawa et al. [21] and Fernández et al. [22], with slight modifications for milk. Briefly, 1 mL of milk and 200 µL of 7.2 % butylated hydroxytoluene were added to a 15-mL conical tube and vortexed. Subsequently, 2 mL of 20 mM thiobarbituric acid and 15 % trichloroacetic acid solutions were added, followed by vortexing. The prepared solutions were incubated at 90 °C for 30 min using a water bath (HB-205SW; Hanbeak Scientific Technology, Gyeonggi-do, Republic of Korea). The mixture was cooled for 10 min before vortexing. Afterward, the samples were centrifuged at 3500 rpm at 4 °C for 15 min. Absorbance was measured at 532 nm using a multimode microplate reader (Varioskan Lux; Thermo Fisher Scientific, MA, USA) with 200 µL of the supernatant in a 96-well plate. The concentration of MDA was calculated using a standard curve generated using 1,1,3,3-tetraethoxypropane, and TBARS values were expressed in milligrams of MDA/L.

#### 2.4.10 Sodium dodecyl sulphate–polyacrylamide gel electrophoresis (SDS-PAGE)

The SDS-PAGE was performed to analyse the molecular weight distribution of milk proteins. Before analysis, the protein concentration of milk samples was adjusted to 1 mg/mL. Samples were then mixed with sample buffer (65.8 mM Tris-HCl, pH 6.8, 26.3 % glycerol, 2.1 % SDS, 0.01 % bromophenol blue, and 5 % 2-mercaptoethanol) in a 1:1 ratio and heated at 95 °C for 5 min, followed by cooling. A total of 20 µL of each prepared milk sample and 10 µL of a pre-stained protein ladder (PageRuler™ Plus Pre-stained Protein Ladder; Thermo Fisher Scientific, MA, USA) were loaded onto a 15 % and 16 % polyacrylamide separating gel, respectively. Electrophoresis was performed at 100 V for 90 min. After separation, the gel was stained with Coomassie Brilliant Blue R-250 staining solution (Bio-Rad, CA, USA) and destained with Coomassie Brilliant Blue R-250 destaining solution (Bio-Rad, CA, USA) until the background was clear.

### 2.5 Statistical analysis

The data were statistically analysed using SPSS software package (Version 29; IBM Corp., Armonk, NY, USA). One-way analysis of variance (ANOVA) was conducted to evaluate statistical differences, followed by Tukey’s multiple comparison test to determine significant mean differences (p < 0.05). All measurements were recorded in triplicate.

## 3 Results and discussion

### 3.1 Microbial analysis

The initial counts of total aerobic bacteria, coliforms, *E. coli* O157:H7, and *L. monocytogenes* in milk samples were 3.90, 2.29, 3.90, and 4.28 log CFU/mL, respectively (Table 1). All of these microorganism counts in milk significantly decreased with increasing focused ultrasound power levels and treatment time. Total aerobic bacteria were not detected after focused ultrasound treatment at 100 W for either 20 or 30 min. Coliforms were not detected by focused ultrasound treatment at 30 W for 30 min, whereas at 100 W for 10, 20, and 30 min. Similarly, no viable *L. monocytogenes* were detected after focused ultrasound treatment at 30 W for 30 min and at 100 W for 20 and 30 min. Focused ultrasound treatment at 100 W for 30 min inactivated all *E. coli* O157:H7 in milk. All microorganism counts in milk significantly decreased after 30 min of heat control treatment compared to 0 min (p < 0.05); however, the reduction was less than 1 log CFU/mL.

**Table 1:** Inactivation effect of microorganisms in milk treated with focused-ultrasound (log CFU/mL).

Microorganism	Time (min)	Heat control^1)^	Focused-ultrasound treatment^2)^		SEM^3)^
			30 W	100 W	
Total aerobic bacteria	0	3.90^A^	3.90^A^	3.90^A^	
	10	3.83^Aa^	3.07^Bb^	2.40^Bc^	0.049
	20	3.80^Aa^	2.89^Bb^	ND^Cc,5)^	0.025
	30	3.23^Ba^	2.84^Bb^	ND^Cc^	0.056
	SEM^4)^	0.044	0.045	0.036	
Coliform	0	2.29^A^	2.29	2.29^A^	
	10	1.88^B^	1.44	ND^B^	0.292
	20	1.81^B^	0.99	ND^B^	0.330
	30	1.77^Ba^	ND^b^	ND^Bb^	0.005
	SEM	0.013	0.330	0.001	
*Escherichia coli* O157:H7	0	3.90^A^	3.90^A^	3.90^A^	
	10	3.89^Aa^	3.74^Aa^	0.81^Bb^	0.269
	20	3.86^Aa^	3.09^Aa^	0.78^Bb^	0.261
	30	3.76^Ba^	0.72^Bb^	ND^Bb^	0.238
	SEM	0.005	0.179	0.281	
*Listeria monocytogenes*	0	4.28^A^	4.28^A^	4.28^A^	
	10	4.20^Ba^	3.77^Aa^	0.74^Bb^	0.249
	20	4.18^Ba^	1.54^Bb^	ND^Bb^	0.257
	30	4.16^Ba^	ND^Bb^	ND^Bb^	0.009
	SEM	0.007	0.194	0.186	

^1)^Raw milk heated at 55 °C.

^2)^Raw milk treated with ultrasound at 55 °C.

^3)^Standard error of means (n = 9), ^4)^(n = 12).

^5)^ND, Not Detected.

^a-c^Values with different letters within the same row differ significantly (p < 0.05).

^A-C^Values with different letters within the same column differ significantly (p < 0.05).

*E. coli* O157:H7 and *L. monocytogenes* are common pathogenic bacteria associated with milk production. *E. coli* O157:H7 is linked to severe illnesses, including haemorrhagic colitis, haemolytic uraemic syndrome, and thrombotic thrombocytopenic purpura, whereas *L. monocytogenes* causes listeriosis, which is a potentially fatal infection [23,24]. Milk contamination with *E. coli* O157:H7 typically occurs via faecal matter from infected cattle, whereas *L. monocytogenes* can enter the milk supply through intramammary infections [25]. Because both pathogens pose a high risk of milk contamination, they should be inactivated. As shown in Table 1, focused ultrasound with mild heat treatment effectively inactivated these pathogens, resulting in an approximate 4 log CFU/mL reduction. Since pathogenic bacteria in raw milk are generally present at 1–10 CFU/mL, and may proliferate up to 10^2^-10^4^ CFU/mL during distribution [26,27], this study evaluated pathogens in milk at levels of approximately 10^4^ CFU/mL. However, further research is needed to achieve a ≥ 5 log reduction, which is critical for the industrial application of focused ultrasound in the milk industry [28].

Ultrasound inactivates microorganisms primarily through acoustic cavitation, which involves the violent implosion of microbubbles. This implosion generates high temperatures and pressures, consequently producing physical effects such as shock waves, shear forces, turbulence, and microjets. These effects collectively disrupt microbial cell membranes by inducing thinning and pore formation, thereby increasing membrane permeability [7,10]. Moreover, they promote intracellular leakage and increase antimicrobial penetration, thereby damaging cellular components such as DNA, proteins, and enzymes. Additionally, cavitation triggers water homolysis to generate free radicals (hydroxyl radicals and hydrogen peroxide) that oxidise microbial membranes, nucleic acids, and proteins, further enhancing their inactivation [10]. Furthermore, Scudino et al. [7] demonstrated that increased nominal ultrasound power enhances microbial inactivation, consistent with the current findings, where the 100 W treatment achieved greater microbial reduction compared to 30 W (Table 1). This enhanced efficacy can be attributed to the increased acoustic cavitation intensity at higher power levels, which promotes greater microbubble formation and, in turn, generates stronger physical forces that contribute to more effective microbial cell disruption.

In the present study, supplementary data provide additional experimental evidence to elucidate the dominant mechanism of microbial inactivation during the focused ultrasound treatment of milk. A potassium iodide assay [29] was conducted to assess the generation of hydroxyl radicals induced by acoustic cavitation (Table S1). Radical generation was higher when focused ultrasound was applied at 20 ℃ than at 55 ℃. However, inactivation of total aerobic bacteria, coliforms, *E. coli* O157:H7, and *L. monocytogenes* was not observed when focused ultrasound was applied at 20 °C (data not shown). This indicated that focused-ultrasound treatment achieved greater bactericidal effects at 55 °C than at 20 °C, despite generating fewer radicals. These results can be explained by the minimal role of radicals generated by ultrasound in microbial inactivation. As shown in Table S2, the acoustic pressure was measured using a Sonicheck-15 device (UL-TECH, Republic of Korea) to evaluate the physical energy delivered by ultrasound. The highest pressure was observed under the optimal milk sterilisation condition (100 W, 55 ℃). This supports the hypothesis that microbial inactivation is predominantly driven by physical rather than chemical mechanisms. In addition, focused-ultrasound treatment showed greater bactericidal effects at 55 °C than at ambient temperatures (22 °C) (data not shown), suggesting that mild heat also contributed to microbial inactivation. According to D'amico et al. [9], the simultaneous action of cavitation and thermal energy disrupts microbial cell membranes, denatures cellular proteins and nucleic acids, and causes oxidative damage to microbial cells via free radicals.

According to Table 1, focused ultrasound at both 30 W and 100 W showed the highest microbial reduction at 30 min. Therefore, physicochemical properties were analysed only for the 30 min focused-ultrasound treatment at 30 W and 100 W and compared with the heat control and LTLT treatment.

### 3.2 ALP activity

**Fig. 1:**
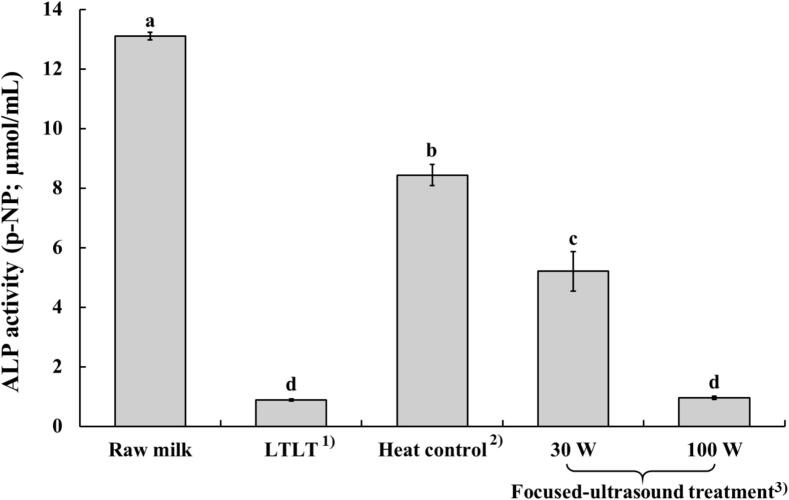
Alkaline phosphatase (ALP) activity of milk treated with focused-ultrasound. ^1)^Raw milk heated at 63 °C for 30 min; ^2)^Raw milk heated at 55 °C for 30 min; ^3)^Raw milk treated with focused-ultrasound at 55 °C for 30 min.

The effect of focused ultrasound on ALP activity is shown in Fig. 1. All treatments reduced ALP activity compared to that observed in raw milk (p < 0.05). LTLT treatment and focused-ultrasound treatment at 100 W exhibited the lowest ALP activity at 0.88 and 0.96 µmol/mL, respectively, and these values were not significantly different (p > 0.05). Focused ultrasound treatment at 30 W showed significantly higher ALP activity (5.21 µmol/mL) than both LTLT and focused ultrasound at 100 W. However, this was significantly lower than that observed for raw milk (8.44 µmol/mL) and heat control (13.10 µmol/mL) (p < 0.05).

ALP is an endogenous hydrolase enzyme naturally present in raw milk. It is widely recognised as a reliable indicator for evaluating the adequacy of pasteurisation [15]. This is due to its slightly greater heat resistance (ALP inactivates between 65 °C and 75 °C) compared to most pathogenic bacteria, including *Mycobacterium tuberculosis*, which is considered the most heat-resistant non-spore-forming pathogen found in raw milk [7,30]. Ultrasound alone, without heat application, shows limited effectiveness against ALP inactivation. However, combining ultrasound with mild heat creates a synergistic effect that can effectively inactivate ALP [7,31]. This indicates that combining focused ultrasound with lower temperatures (55 °C) enables effective ALP inactivation, offering a viable alternative to conventional heat treatments (such as LTLT), a finding supported by the current research.

### 3.3 IgG retention

**Fig. 2:**
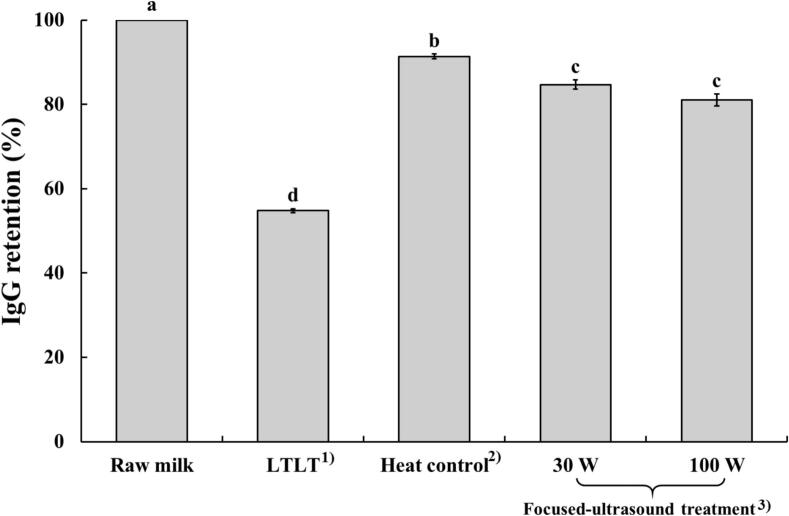
Immunoglobulin G (IgG) retention in milk treated with focused ultrasound. ^1)^Raw milk heated at 63 °C for 30 min; ^2)^Raw milk heated at 55 °C for 30 min; ^3)^Raw milk treated with focused-ultrasound at 55 °C for 30 min.

IgG retention for each milk sample following ultrasound and heat treatments is expressed as a percentage relative to the IgG concentration in raw milk (Fig. 2). LTLT, heat control, and focused ultrasound treatments significantly reduced IgG retention compared to raw milk (p < 0.05). The lowest IgG retention was observed in the LTLT treatment (54.78 %). Furthermore, focused ultrasound treatments at 30 W (84.66 %) and 100 W (81.00 %) resulted in higher IgG levels than LTLT treatment (p < 0.05). No significant difference in IgG retention was observed among the focused ultrasound treatments at different power levels. The heat control treatments showed lower IgG retention (91.39 %) than raw milk, but higher retention than both focused ultrasound treatments (p < 0.05).

Bovine IgG prevents the adhesion of pathogens to epithelial surfaces and facilitates the uptake of IgG pathogen immune complexes via Fc receptors in the human small intestine, thereby enhancing phagocytosis and triggering a regulatory immune response. In the colon, bovine IgG contributes to the modulation of the gut microbial composition, reduces lipopolysaccharide translocation across the intestinal barrier, and influences the production of short-chain fatty acids that eventually improve immunity [32]. The IgG reduction observed in ultrasound-treated milk compared to raw milk in the present study was similar to that reported by Chen et al. [2] and Liu et al. [8]. This inactivation was primarily attributed to the physical forces generated by the acoustic streaming induced by ultrasound-mediated cavitation. The rapid formation and collapse of cavitation bubbles during ultrasound treatment generate intense localised shear forces. These forces can disrupt hydrogen bonds and van der Waals interactions, leading to alterations in the secondary and tertiary structures of IgG, resulting in the loss of its biological activity [33]. In addition, the extent of IgG denaturation in milk increases with rising temperature [34]. Our results support this notion, where the comparatively higher temperature in the LTLT (63 °C) process likely contributed to the lowest IgG retention among the tested treatments, especially compared to ultrasound treatments (55 °C). These results suggest that ultrasound is a promising alternative to LTLT for preserving IgG levels in milk at lower temperatures.

### 3.4 Particle size and particle size distribution

**Fig. 3:**
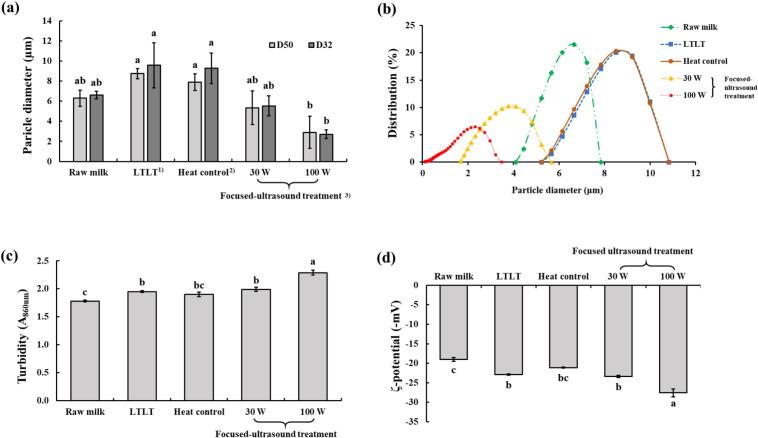
(a) Particle size, (b) particle size distribution, (c) turbidity, and (d) ζ-potential in milk treated with focused ultrasound. ^1)^Raw milk heated at 63 °C for 30 min; ^2)^Raw milk heated at 55 °C for 30 min; ^3)^Raw milk treated with focused-ultrasound at 55 °C for 30 min.

Fig. 3 (a) shows the median particle diameter (D_50_) and surface mean diameter (D_32_) of the milk samples. The D_50_ and D_32_ values were the lowest in milk treated with focused ultrasound at 100 W (2.9 and 2.71 µm, respectively), whereas the highest values were observed for LTLT treatment (8.75 and 9.57 µm) and heat control (7.90 and 9.57 µm) (p < 0.05). The D_50_ and D_32_ values of focused-ultrasound treatment at 30 W were 5.34 and 5.53 µm, respectively. These values were not significantly different from those observed for raw milk (6.30 and 6.62 µm).

Fig. 3 (b) shows the particle size distribution of milk samples. The distribution curves of the LTLT and heat control treatments shifted towards the right, indicating an increase in particle size compared with that of raw milk. In contrast, the distribution curves corresponding to the focused ultrasound treatments at 30 W and 100 W shifted to the left, signifying a reduction in particle size. Moreover, the focused ultrasound treatment at 100 W resulted in the smallest particle size distribution among all treatments and shifted most to the left in the distribution curve.

Particle size distribution, particularly milk fat globule size distribution, plays a crucial role in determining the physical stability of milk fat emulsions. Increasing ultrasound power enhances the formation of cavitation bubbles, and their subsequent implosion generates more intense hydrodynamic shear forces that act upon milk constituents (particularly milk fat globules), resulting in a reduction in their size [35]. This effect was evident in the present study, as demonstrated by the D_32_ and D_50_ values, which showed a significant reduction in particle size in the ultrasound 100 W treatment compared to LTLT.

Generally, following the rupture of the milk fat globule, caseins and whey proteins adsorb onto the newly formed milk fat globule membrane, consequently contributing to emulsion stability by preventing coalescence and creaming [36]. However, higher ultrasound power levels (higher energy input) can increase the fat globule disruption rate beyond the rate at which caseins and whey proteins are adsorbed onto the newly formed milk fat globule membranes [7]. This imbalance promotes the recoalescence of fat globules, leading to an increase in particle size. Nonetheless, in the present study, the ultrasound 100 W treatment did not cause a larger fat globule size than the 30 W treatment, suggesting that the milk remained stable under 100 W treatment conditions. In the present study, the larger particle size observed in the LTLT treatment may be attributed to its elevated processing temperature (63 °C) compared to that of other treatments conducted at 55 °C. Elevated temperatures denature milk fat globule membrane proteins, thereby disrupting the electrolyte balance and enhancing the mobility of fat globules, which promotes flocculation and coalescence, ultimately increasing the particle size [37]. The particle distribution is also consistent with these explanations. Because ultrasound treatments reduce particle size, the curves of ultrasound at 100 W and 30 W shifted towards the left side, a pattern also reported by Scudino et al. [7], who showed that the ultrasound curve shifted the smaller particle size area away from the raw milk.

### 3.5 Turbidity

As shown in Fig. 3 (c), the turbidity value was the lowest in raw milk (1.78), and it was not significantly different from that of the heated control (1.90). Focused ultrasound treatment at 100 W resulted in the highest turbidity (2.29; p < 0.05). Focused ultrasound treatments at 30 W (1.98) and LTLT (1.95) showed no significant differences in turbidity (p > 0.05).

**Fig. 4:**
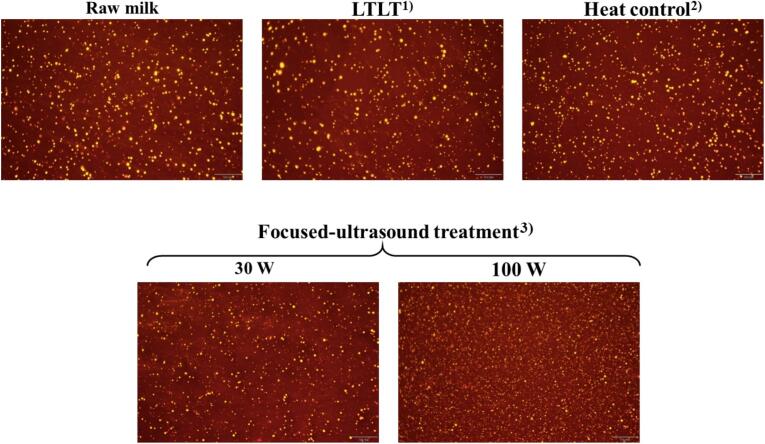
Micrographs of fat globules in milk treated with focused-ultrasound. ^1)^Raw milk heated at 63 °C for 30 min; ^2)^Raw milk heated at 55 °C for 30 min; ^3)^Raw milk treated with focused-ultrasound at 55 °C for 30 min.

Turbidity can be indicated by the light scattering from an emulsion. Ultrasound induces protein dissociation and aggregation, thereby increasing the turbidity of bovine milk [38], which aligns with our observations. Furthermore, in the present study, high-power focused ultrasound effectively disrupted fat globules into smaller particles, which were more uniformly distributed compared to other treatments (see Fig. 4). This enhanced dispersion likely contributed to the increased turbidity observed which further supported by Dahm [39], who reported that a higher number of dispersed particles in milk enhanced light scattering and subsequently increased turbidity.

### 3.6 Zeta potential

As shown in Fig. 3 (d), all treated milk samples exhibited reduced zeta potential compared with raw milk (p < 0.05). Focused ultrasound treatment at 100 W showed the most negative zeta potential value (−27.6 mV; p < 0.05). LTLT, heat control, and focused ultrasound treatment at 30 W exhibited lower zeta potential (p < 0.05), with values of –22.91, −21.12, and –23.38 mV, respectively, compared to ultrasound 100 W.

The zeta potential is an important indicator of emulsion stability. A higher absolute value of the zeta potential indicates stronger electrostatic repulsion among particles, which prevents aggregation and enhances emulsion stability in milk [40]. The zeta potential of milk is associated with the stability of both casein micelles and milk fat globules, as both components generally carry a net negative surface charge [9]. Ultrasound-induced cavitation can modify milk fat globule membrane protein structures and alter the orientation of phospholipid head groups (particularly phosphatidylcholine) on the membrane surfaces, which could increase the negative zeta potential of milk [40]. Furthermore, Jukkola et al. [41] reported that ultrasound treatment increased the negative zeta potential of milk fat globules, likely due to cavitation-induced damage to the milk fat globule membrane and the adsorption of negatively charged casein proteins onto the globule surface. As the 100 W ultrasound treatment resulted in the most negative zeta potential value in the present study, this indicates that milk treated at 100 W exhibited the highest stability among all the treatments.

### 3.7 Microstructure of the fat globules

The effects of focused ultrasound and heat treatment on the microstructure of milk fat globules are shown in Fig. 4. Fat globules in raw milk appeared visibly larger than those in focused ultrasound-treated milk. Among all the treatments, focused ultrasound at 100 W resulted in the smallest fat globule size, followed by ultrasound at 30 W. In contrast, LTLT and heat control treatments exhibited relatively larger fat globules than raw milk, with the LTLT treatment showing the largest overall fat globule size.

The microstructural images support the droplet size and distribution results observed in the present study, where raw, LTLT, and heat-control milk samples exhibited larger fat globules than the ultrasound-treated milk samples. During ultrasound processing, the larger fat globules in raw milk were effectively reduced in size owing to cavitation effects, which fragmented them into smaller droplets. Among the treatments, 100 W ultrasound produced the most uniform and smallest fat globule distribution. These findings are consistent with the study by Scudino et al. [7], who reported that a higher nominal ultrasound power led to a greater reduction in milk fat globule size than that in untreated raw milk. Furthermore, microstructural image observations in the present study confirmed that the combined application of ultrasound and heat treatment reduced the milk fat globule size distribution, with a progressive decrease observed as ultrasonication exposure time and treatment temperature increased [42].

### 3.8 Colour values

In Table 2, LTLT, heat control, and focused ultrasound treatments did not affect the L* and a* values, and the whiteness index of milk (p > 0.05). Nevertheless, the b* and ΔE values of milk increased following focused ultrasound treatment at 100 W compared to those of raw milk and milk subjected to other treatments (p < 0.05). The ultrasound-induced acoustic cavitation enhances milk homogenisation by reducing particle size, which increases light scattering and consequently elevates the L* value of milk; this has been well-documented in previous studies [2,7,43]. However, a similar effect was not observed in the present study. Ultrasound-assisted emulsification disrupts milk fat globules, consequently facilitating the release and encapsulation of embedded β-carotene and reducing its degradation. This eventually contributes to an increase in the b* colour parameter of whole milk [44,45]. Additionally, ultrasound-induced cavitation inactivates lactoperoxidase (an oxidoreductase enzyme), thereby protecting β-carotene from enzymatic degradation and enhancing the b* value in milk [46]. Furthermore, the combination of heat and ultrasound may promote the Maillard reaction, potentially contributing to an increase in the yellowness of milk and dairy products. This is likely due to the formation of melanoidins (brownish-yellow pigments) generated during the Maillard reaction, which impart a yellowish hue to the product [7,47,48].

**Table 2:** Color, pH, and thiobarbituric acid reactive substances (TBARS) value of milk treated with focused-ultrasound.

Quality parameter	Raw milk	LTLT^1)^	Heat control^2)^	Focused-ultrasound treatment^3)^		SEM^4)^
				30 W	100 W	
CIE color values						
L*	58.52	58.60	58.54	58.66	58.69	0.046
a*	1.16	1.16	1.16	1.15	1.15	0.002
b*	17.77^b^	17.92^b^	17.86^b^	18.04^b^	18.36^a^	0.026
ΔE	0.00^c^	0.21^bc^	0.36^b^	0.30^b^	0.66^a^	0.024
Witness Index	54.86	54.87	54.84	54.88	54.77	0.040
pH	6.72	6.73	6.73	6.72	6.72	0.001
TBARS (mg MDA/L)	0.035^d^	0.073^bc^	0.064^c^	0.081^ab^	0.085^a^	0.001

^1)^Raw milk heated at 63 °C for 30 min

^2)^Raw milk heated at 55 °C for 30 min

^3)^Raw milk treated with focused-ultrasound at 55 °C for 30 min

^4)^Standard error of mean (n = 15).

^a-d^Values with different letters within the same row differ significantly (p < 0.05).

Although the 100 W ultrasound treatment resulted in the highest ΔE value among all treatments in the present study, it remained below one (ΔE < 1). This indicated no noticeable overall colour change, as a ΔE value below one is generally considered imperceptible to the human eye. Similarly, the whiteness index did not differ significantly across treatments, suggesting that ultrasound combined with mild heat processing did not alter the degree of whiteness of milk [20].

### 3.9 pH and TBARS values

The pH values were not significantly different across all the treatment groups (p > 0.05; Table 2). The pH is a critical indicator of milk quality. Fresh milk typically has a pH of approximately 6.7. Casein, the main protein in milk, remains evenly dispersed under normal pH conditions. However, a decrease in pH promotes a decrease in casein levels and the release of free fatty acids. Furthermore, it contributes to the development of off-flavours. Thus, maintaining milk pH is important for the sensory acceptability and quality of milk [49,50]. Our results are similar to those of Pegu and Arya [51], who reported a statistically insignificant reduction in milk pH across different ultrasound power levels. However, our results contradict the findings of Scudino et al. [7], who reported that ultrasound treatments could decrease the pH of milk attributed to lipolysis resulting from the enzymatic action on triglycerides. This enzymatic activity may be facilitated by the generation of reactive oxygen species such as nitrite, nitrate, and hydrogen peroxide during ultrasound treatment.

MDA, a secondary product of lipid peroxidation, is generated through the oxidation of polyunsaturated fatty acids (PUFAs) and can indicate lipid oxidation levels in food systems [52]. Table 2 presents the TBARS values in milk, measured as MDA concentration (mg/L). Compared to raw milk, LTLT, heat control, and focused-ultrasound treated milk exhibited significantly higher TBARS values (p < 0.05). The focused ultrasound treatment at 100  W resulted in the highest TBARS value (0.085  mg MDA/L), which was 0.012  mg MDA/L higher than that of the LTLT treatment (0.073  mg MDA/L). Ultrasound treatment promotes lipid oxidation via acoustic cavitation in milk, consequently generating free radicals that trigger chain reactions in PUFAs and forming unstable hydroperoxides that degrade into aldehydes and ketones. This treatment also disrupts milk fat globule membranes, thereby exposing lipids to oxidative damage [52]. Similar to our results, an increase in temperature elevates TBARS values, reflecting higher levels of lipid oxidation [2]. Elevated temperatures accelerate the breakdown of PUFAs during thermal processing, leading to the formation of reactive species such as peroxyl radicals and lipid alkyl radicals. These radicals further react with unsaturated fatty acids and promote the lipid oxidation process [2]. Consequently, the combined effects of temperature and ultrasound resulted in increased lipid oxidation in ultrasound-treated milk compared to the other treatments in our study. However, Johnson et al. [53] suggested a TBARS threshold of 1.3 mg MDA/L as the sensory limit for perceivable oxidation of milk. In the present study, TBARS values remained below this threshold, suggesting that the focused-ultrasound treatment at 55 °C did not compromise the sensory quality of the milk and was within acceptable oxidative limits. However, for industrial applications, future studies should include sensory evaluation of milk treated with focused ultrasound for industrial applications.

### 3.10 SDS-PAGE

**Fig. 5:**
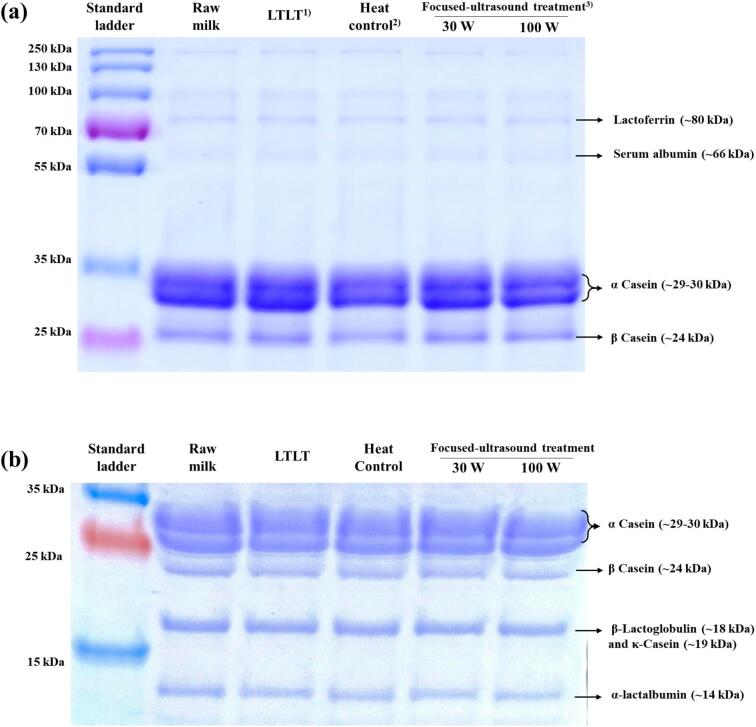
Electrophoretic banding patterns obtained under SDS-PAGE in milk treated with focused ultrasound. (a) 15 % separating gel; (b) 16 % separating gel. ^1)^Raw milk heated at 63 °C for 30 min; ^2)^Raw milk heated at 55 °C for 30 min; ^3)^Raw milk treated with focused-ultrasound at 55 °C for 30 min.

Fig. 5 (a) and (b) show the electrophoretic banding patterns of milk samples obtained by SDS-PAGE. In raw milk, heat control, LTLT, and focused ultrasound-treated milk at 30 and 100 W, distinct protein bands corresponding to α-casein (∼ 29–30 kDa), β-casein (∼24 kDa), κ-casein (∼19 kDa), β-lactoglobulin (∼18 kDa), α-lactalbumin (∼14 kDa), lactoferrin (∼80 kDa), and serum albumin (∼66 kDa) were observed. None of the treatments showed any indication of additional or loss of protein bands. These results suggest that functional and nutritional protein components were not degraded or aggregated after focused ultrasound processing, which is critical for ensuring the overall milk quality.

Milk proteins are fundamental to human nutrition; they serve as rich sources of essential amino acids and various bioactive compounds [54]. Casein proteins in milk exhibit anticarcinogenic properties, particularly against colon cancer, by modulating immune responses, enhancing phagocytic activity, and increasing lymphocyte proliferation. Specifically, α-casein facilitates calcium and phosphate transport to improve nutrient absorption. Similarly, β-casein lowers blood cholesterol, whereas κ-casein exhibits anticarcinogenic effects by inhibiting plaque-forming enzymes [1]. β-Lactoglobulin exhibits anticarcinogenic effects by stimulating glutathione synthesis. Furthermore, it shows antiviral activity while serving as a carrier for fat-soluble nutrients such as vitamin A [1,55]. α-Lactalbumin possesses several bioactive properties, including antibacterial and antiviral activities. It also provides a balanced amino acid profile, which is important for infant nutrition [1,56]. Lactoferrin contributes to immunomodulation by enhancing delayed-type hypersensitivity responses to diverse antigens. It also exhibits anti-carcinogenic effects, which can be attributed to its anti-inflammatory and antioxidant properties. Moreover, lactoferrin has protective effects against alcohol-induced chronic liver damage [1]. Milk serum albumin also facilitates the transport of fatty acids across cell membranes, thereby supporting lipid metabolism and cellular energy production [57]. As shown in Fig. 5, these milk proteins were neither aggregated nor degraded by focused ultrasound treatment, indicating that the functional and protein properties of milk were not significantly altered.

Consistent with our observations, Sun et al. [58] detected no differences in protein band patterns or molecular weight between untreated and ultrasound-treated milk, confirming the integrity of the major protein structures in milk (caseins and whey proteins). Furthermore, Zhang et al. [33] observed no loss of protein bands for key functional proteins such as immunoglobulin, β-lactoglobulin, and lactoferrin following ultrasound treatment. Similarly, SDS-PAGE analysis by Liu et al. [8] demonstrated that the major serum protein bands of whole milk remained unchanged, indicating that ultrasound treatment did not compromise the molecular structure of milk proteins.

## 4 Conclusion

The present study confirmed that focused ultrasound treatment (at 55 °C and 100  W for 30 min) effectively inactivated total aerobic bacteria, coliforms, *E. coli* O157:H7, and *L. monocytogenes* in milk. Furthermore, this treatment resulted in a significant reduction in ALP activity. These results indicate that focused ultrasound achieved effective microbial inactivation at a temperature 8 °C lower than that for conventional LTLT pasteurisation (63 °C). In addition, focused ultrasound treatment preserved higher IgG activity than LTLT, indicating its potential for enhancing the health benefits of milk. The smaller particle size and negatively higher zeta potential values observed in focused ultrasound-treated milk, compared to LTLT milk, suggest improved fat dispersion and emulsion stability. Focused ultrasound treatment slightly increased lipid oxidation and the b* value of milk but did not affect the L* and a* values, whiteness index, pH, and the molecular weight of milk proteins (as confirmed by SDS-PAGE analysis). These results indicate that the thermo-cylindrical-type focused ultrasound system can improve microbial safety, IgG retention, and fat dispersion with minimal quality changes. This focused ultrasound system shows potential as a promising novel milk pasteurisation technology. In addition, a pilot-scale production model (DEBREX 500) incorporating the performance of the research system has been developed, with a demonstrated processing capacity of up to a few hundred liters per day. Ongoing scale-up studies are further evaluating its feasibility for industrial application. However, further research is still required to investigate the nutrient retention and sensory properties of focused ultrasound-treated milk for industrial applications.

## Author contributions

• Prabhathma Yasasvi Rathnayake and Soyeong Kim wrote the main manuscript text.
• Rina Yu and Chemin Nam conducted the experiments and analyzed the data.
• Sin-Young Park prepared the figures and tables.
• Seonae Hwangbo and Hae In Yong conceptualized and supervised the study
• All authors reviewed and approved the final manuscript.

## CRediT authorship contribution statement

**Prabhathma Yasasvi Rathnayake:** Writing – review & editing, Writing – original draft, Investigation. **Soyeong Kim:** Writing – review & editing, Writing – original draft. **Rina Yu:** Methodology, Investigation, Data curation. **Chemin Nam:** Methodology, Investigation, Data curation. **Sin-Young Park:** Investigation. **Seonae Hwangbo:** Supervision, Funding acquisition, Conceptualization. **Hae In Yong:** Supervision, Project administration, Methodology, Investigation, Conceptualization.

## Declaration of competing interest

The authors declare the following financial interests/personal relationships which may be considered as potential competing interests: Seonae Hwangbo reports financial support was provided by Commercialization Promotion Agency for R&D Outcomes (COMPA). Seonae Hwangbo reports financial support was provided by Korea Evaluation Institute of Industrial Technology (KEIT). Seonae Hwangbo reports financial support was provided by Ministry of Science and ICT (MSIT) of the Republic of Korea. Hae In Yong reports financial support was provided by National Research Foundation of Korea (NRF) grant funded by the Korea government (MSIT). If there are other authors, they declare that they have no known competing financial interests or personal relationships that could have appeared to influence the work reported in this paper.
