# Supplementary material for: Application of thermo-cylindrical type focused-ultrasound as novel milk pasteurization: microbial inactivation, immunoglobulin G retention, and physicochemical characteristics
Source: Ultrason Sonochem. 2025 Oct 23;123:107615. doi: 10.1016/j.ultsonch.2025.107615 (PMC12605656; doi:10.1016/j.ultsonch.2025.107615)
Supplement: Supplementary Data 1 [file mmc1.docx]

**Supplementary Data**

**Table S1.** Effect of various focused-ultrasound treatments on potassium iodide-based radical detection level

| **Frequency**  **(kHz)** | **Power**  **(W)** | **Temperature**  **(****℃)** | **Time**  **(min)** | **Absorbance**  **(A.U.)** |
| --- | --- | --- | --- | --- |
| 400 | 30 | 55 | 30 | 0.113 |
| 400 | 100 | 55 | 30 | 0.192 |
| 400 | 30 | 20 | 30 | 0.257 |
| 400 | 100 | 20 | 30 | 0.852 |

**Table S2.** Effect of various focused-ultrasound treatments on acoustic pressure

| **Frequency**  **(kHz)** | **Power**  **(W)** | **Temperature (℃)** | **Acoustic Pressure  (mV, relative)** |
| --- | --- | --- | --- |
| 400 | 30 | 55 | 81.9 |
| 400 | 100 | 55 | 221.0 |
| 400 | 30 | 20 | 49.9 |
| 400 | 100 | 20 | 168.6 |
